# Utilizing protein structure graph embeddings to predict the pathogenicity of missense variants

**DOI:** 10.1093/nargab/lqaf097

**Published:** 2025-07-24

**Authors:** Martin Danner, Matthias Begemann, Miriam Elbracht, Ingo Kurth, Jeremias Krause

**Affiliations:** Institute for Human Genetics and Genomic Medicine, Medical Faculty, Uniklinik RWTH Aachen, Pauwelsstrasse 30, Aachen 52074, North-Rhine-Westphalia, Germany; scieneers GmbH, Data Science Department, Kantstraße 1a, Karlsruhe 76137, Baden-Wuerttemberg, Germany; Institute for Human Genetics and Genomic Medicine, Medical Faculty, Uniklinik RWTH Aachen, Pauwelsstrasse 30, Aachen 52074, North-Rhine-Westphalia, Germany; Institute for Human Genetics and Genomic Medicine, Medical Faculty, Uniklinik RWTH Aachen, Pauwelsstrasse 30, Aachen 52074, North-Rhine-Westphalia, Germany; Institute for Human Genetics and Genomic Medicine, Medical Faculty, Uniklinik RWTH Aachen, Pauwelsstrasse 30, Aachen 52074, North-Rhine-Westphalia, Germany; Institute for Human Genetics and Genomic Medicine, Medical Faculty, Uniklinik RWTH Aachen, Pauwelsstrasse 30, Aachen 52074, North-Rhine-Westphalia, Germany

## Abstract

Genetic variants can impact the structure of the corresponding protein, which can have detrimental effects on protein function. While the effect of protein-truncating variants is often easier to evaluate, most genetic variants that affect the protein-coding region of the human genome are missense variants. These variants are mostly single nucleotide variants, which result in the exchange of a single amino acid. The effect on protein function of these variants can be challenging to deduce. To aid the interpretation of missense variants, a variety of bioinformatic algorithms have been developed, yet current algorithms rarely directly use the protein structure as a feature to consider. We developed a machine learning workflow that utilizes the protein-language-model ESMFold to predict the protein structure of missense variants, which is subsequently embedded using graph autoencoders. The generated embeddings are used in a classifier model, which predicts pathogenicity. We provide evidence that graph embeddings can be used for pathogenicity prediction and that they can be used to enhance the widely applied CADD score. Additionally, we explored different levels of abstraction of the graph embeddings and their influence on the classifier. Finally, we compare the utility of graph embeddings from different protein-folding models.

## Introduction

Rare diseases, while individually uncommon, collectively affect ∼5% of the global population, or ∼350 million people [1]. With around 70% of these cases involving children, the importance of early diagnosis and treatment cannot be overstated [1]. However, the current average diagnosis time still stands at a distressing 5–7 years [1]. Roughly 80% of these rare diseases have a genetic cause [1]. Given the complexity of our genome, with its 3.3 billion bases and each individual carrying ∼3.7 million variants and ∼10.000 non-synonymous variants within the protein-coding region, pinpointing the single disease-causing variant is extremely challenging [2]. This is further complicated by the fact that for the majority of known missense variants, a frequent cause of disease, the impact on protein function is unclear, and the variants are therefore classified as variants of unknown clinical significance [3]. This frequently leads to inconclusive results when genetic testing is performed in a clinical setting [4]. To better characterize missense variants and to eventually enter an age without variants of unknown clinical significance, different strategies have been proposed [5]. Ultimately, experimental characterizations might be necessary to achieve this state. However, experimental approaches are resource and time consuming and consequently don’t offer promises for scalability in the near future [6]. Therefore, different bioinformatic prioritization strategies have been proposed to narrow down the number of candidates for experimental follow-up [7–18]. Especially machine learning models offer a promising avenue for enhancing the process of detecting and prioritizing variants [6, 19]. Pathogenicity prediction models typically include a range of genomic features, which are aggregated and delivered to a statistical or machine learning model that performs a classification or regression task [8]. These features can include population metrics (e.g. the population allele frequency of a variant), evolutionary conservation metrics, the sequence context, and epigenetic data. However, despite the progress in computational prediction strategies (e.g. AlphaFold [20]), three-dimensional data are rarely used. Current models that use this information are either computationally expensive and bound to structure predictions from specific models (AlphaMissense [6]) or do not use the structure itself but singular and independent information derived from this structure (SIGMA [21], AlphScore [22]). Furthermore, in the case of AlphaMissense and AlphScore, the models are only presented with wild-type structures during training [6, 22]. While it has been previously demonstrated that variant structures predicted by AlphaFold2 don’t always agree with experimental data [23], models like SIGMA highlight the potential of *in silico* predicted variant structures [21]. In this study, we introduce a novel machine learning approach that directly leverages information from *in silico* predicted protein structures of missense variants and their corresponding wild-type structures. Importantly, we used ESMFold [24] to predict over 60 000 protein structures to aid this process. We demonstrate the practicality and value of using *in silico* predicted protein structures, such as those modeled by ESMFold [24], both as an independent pathogenicity predictor and as an additional feature that is especially valuable for enriching established pathogenicity prediction scores, e.g. when combined with the widely used CADD score. This study further highlights the potential of machine learning in aiding the diagnosis of rare diseases.

## Materials and methods

### Materials

ProteinGym is a large-scale dataset published by Notin *et al.*that serves as a benchmark for protein design models and fitness predictions, aiming to establish a basis for comparison across different studies [25]. It contains aggregated deep mutational scanning assays and a smaller clinical dataset, derived from expert-curated variant collections. Both sets are available for missense and indel variants. Additionally, it provides a benchmark board, where different pathogenicity prediction tools are ranked. For this study, we utilized the clinical substitution dataset, which contains 63 914 missense variants. The dataset consists of 31 546 variants that are classified as benign and 32 638 variants ranked as pathogenic variants. The included variants affect 2525 genes in total.

The AlphaFold database contains over 214 million predicted protein structures as of 2024 [26]. The structures were predicted using the AlphaFold2 model from DeepMind. We utilized the ID mapping feature from UniProt [27] to map the proteins present in ProteinGym (identified by the NCBI protein ID) to prefolded structures from the AlphaFold database. This mapping was successful for 2411 unique proteins present in ProteinGym, which translated to 59 525 out of 63 914 variants. Experiments containing the AlphaFold2 structures were therefore limited to this subset of ProteinGym.

The CADD [19] score is a pathogenicity prediction score that is widely used to rank genetic variants in the context of pathogenicity prediction, especially for the identification of Mendelian monogenic diseases. It integrates a variety of features, such as conservation metrics (among other scores, the phyloP score from the Zoonomia [28] project), information about epigenetic modifications, effects on protein function, and the genomic context [19]. In its latest iteration, among other things, it also includes information derived from the ESM protein language model [24]. The CADD score is precomputed for the whole human genome and therefore offers a wide range of applications, especially in the context of rare diseases. We obtained precomputed CADD scores for the ProteinGym dataset by transforming the clinical substitution dataset from ProteinGym into a singular variant call format file, which was subsequently annotated using the CADD annotation service hosted on the CADD website. In this process, a few variants were unmappable, which translated to 59 998 out of 63 914 variants from 2406 unique proteins. All experiments in which the CADD score was used or in which a classifier was compared to a classifier utilizing the CADD score were therefore limited to this subset.

## Methods

**Figure 1.**
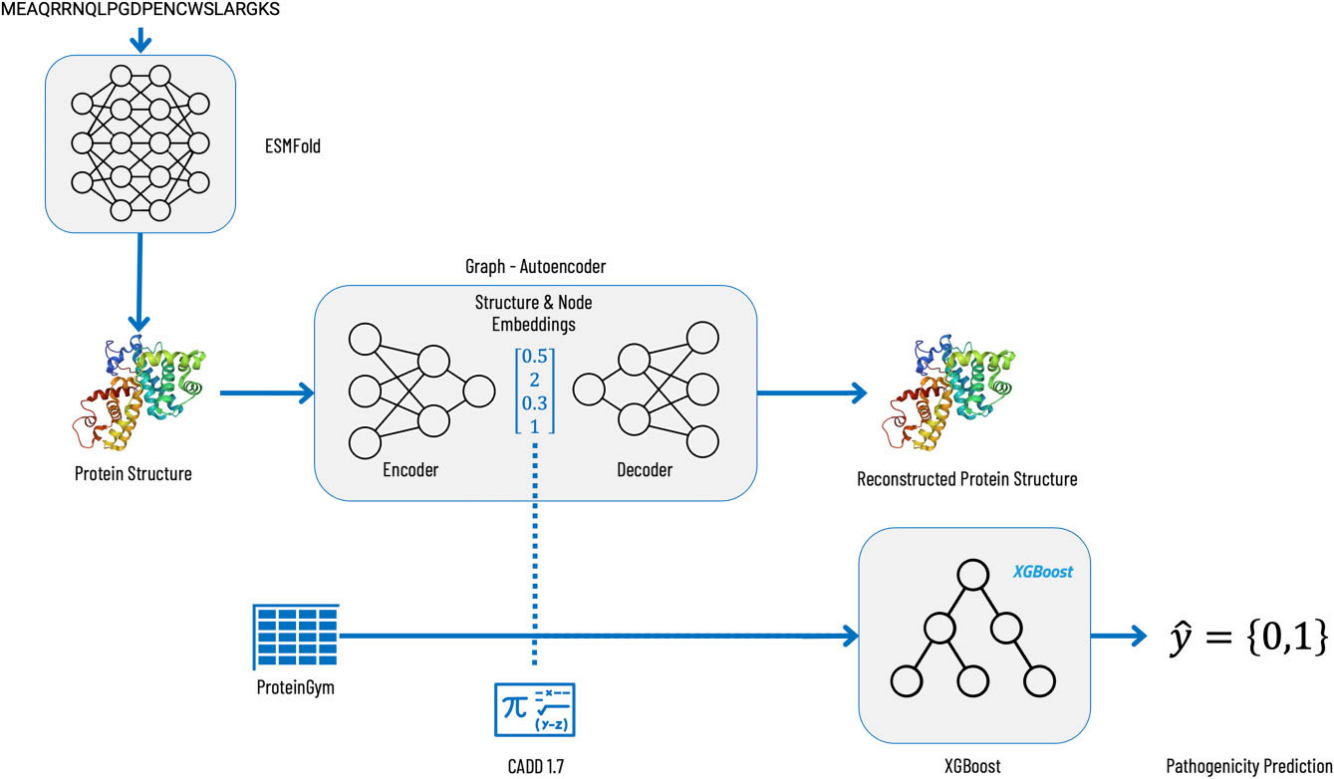
Schematic illustration of the machine learning approach workflow. We predicted protein structures for 59 525 missense single nucleotide variants and their corresponding wild types using ESMFold. These structures were then converted into numerical representations, so called embeddings, via a GAEs. Finally, an XGBoost classifier was trained using these embeddings either on their own or in combination with the CADD score.

The presented workflow started with a protein language model. ESMFold was used to generate *in silico* predictions for the protein structures of the wild types and structures of the variants contained in the clinical substitution dataset from ProteinGym. ESMFold was selected because it provides a much faster folding time and is more portable when compared to AlphaFold2, although it comes with slightly less accurate predictions. The generated structures were used to train graph autoencoders (GAEs) [29] in order to generate structural embeddings for both the wild types and their corresponding variants. These structural embeddings were then either used on their own or combined with the CADD score. The selected features were finally used to train an XGBoost [30] model on a binary classification task to predict the pathogenicity of missense single nucleotide variants. The complete workflow is detailed in Fig. 1.

### Protein structure prediction with ESMFold

To generate the protein structures, we employed the ESMFold model (3B) from Hugging Face [31], which was utilized to predict the protein structures for both the variants and corresponding wild types within the ProteinGym dataset. This process was conducted on multiple virtual machines within the Azure Machine Learning environment, each equipped with an A100 GPU.

Given the O(n^3^) complexity of the ESMFold model, attempting to run inference on larger amino sequences resulted in memory overflow issues [24]. To circumvent this, we divided longer sequences into smaller subsequences, running the inference on each subsequence individually. The individual predictions were subsequently stitched together in a post-processing step using the NumPy library. The resulting protein structures were stored as Protein Data Bank [32] files for subsequent processing and analysis in our study. This strategy allowed us to efficiently manage computational resources while generating a comprehensive set of protein structure predictions for our machine learning approach.

### Preparation of protein graph datasets

**Figure 2.**
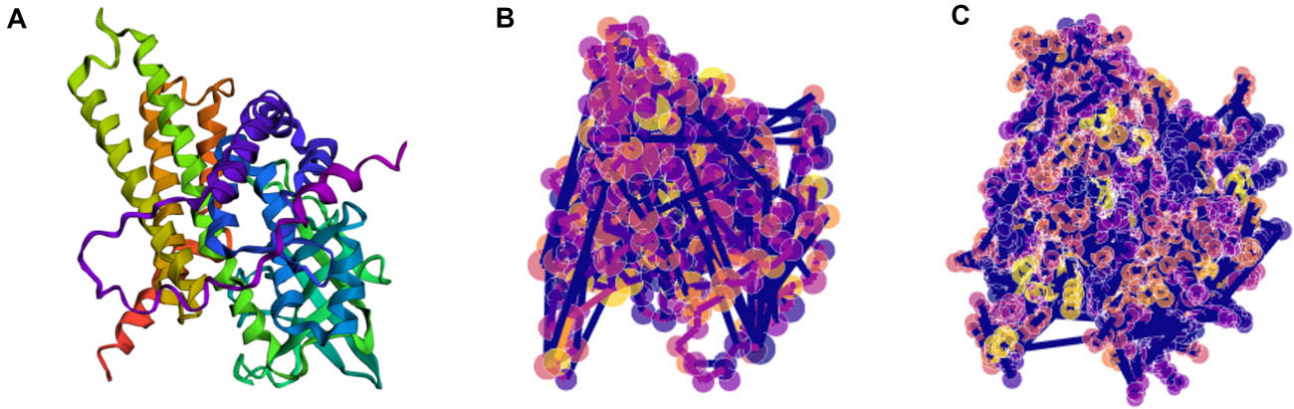
Protein structure of A113D (**A**) consisting of 421 amino acids and its conversion in a residue-scoped graph representation (**B**) with 421 nodes and 1136 edges, as well as its conversion in an atomic-scoped graph representation (**C**) with 3274 nodes and 3338 edges. Conversion was carried out using Graphein.

We transformed the predicted protein structures from ESMFold into graph datasets represented as PyTorch Geometric Objects [33] using Graphein [34]. In more detail two distinct graph datasets were generated to extract structural embeddings of varying scopes, as shown in Fig. 2:

- An atomic-scoped dataset, where individual atoms are represented by each node, and covalent edges are based on atomic distances. The node features included the 3D coordinates and a one-hot encoding of the atom. The min-max scaled atomic distances were also included as edge features.
- A residue-scoped dataset, where each node represents a residue in turn depicted by the alpha carbon and edges signify various interactions—distance based, aromatic, hydrogen bond, hydrophobic, aromatic sulphur, disulfide, cation pi, and peptide bonds. Node features included the 3D coordinates, the one-hot encodings of the amino acid, and details about the residue’s presence of a hydrogen bond acceptor/donor.

Both graph datasets, containing wild-type and variant structures, were divided into subsets for training, validation, and testing of the GAEs. The division was conducted at the level of individual structures in a 70% (training), 20% (validation), and 10% (test) ratio.

### Structural embeddings with GAEs

**Figure 3.**
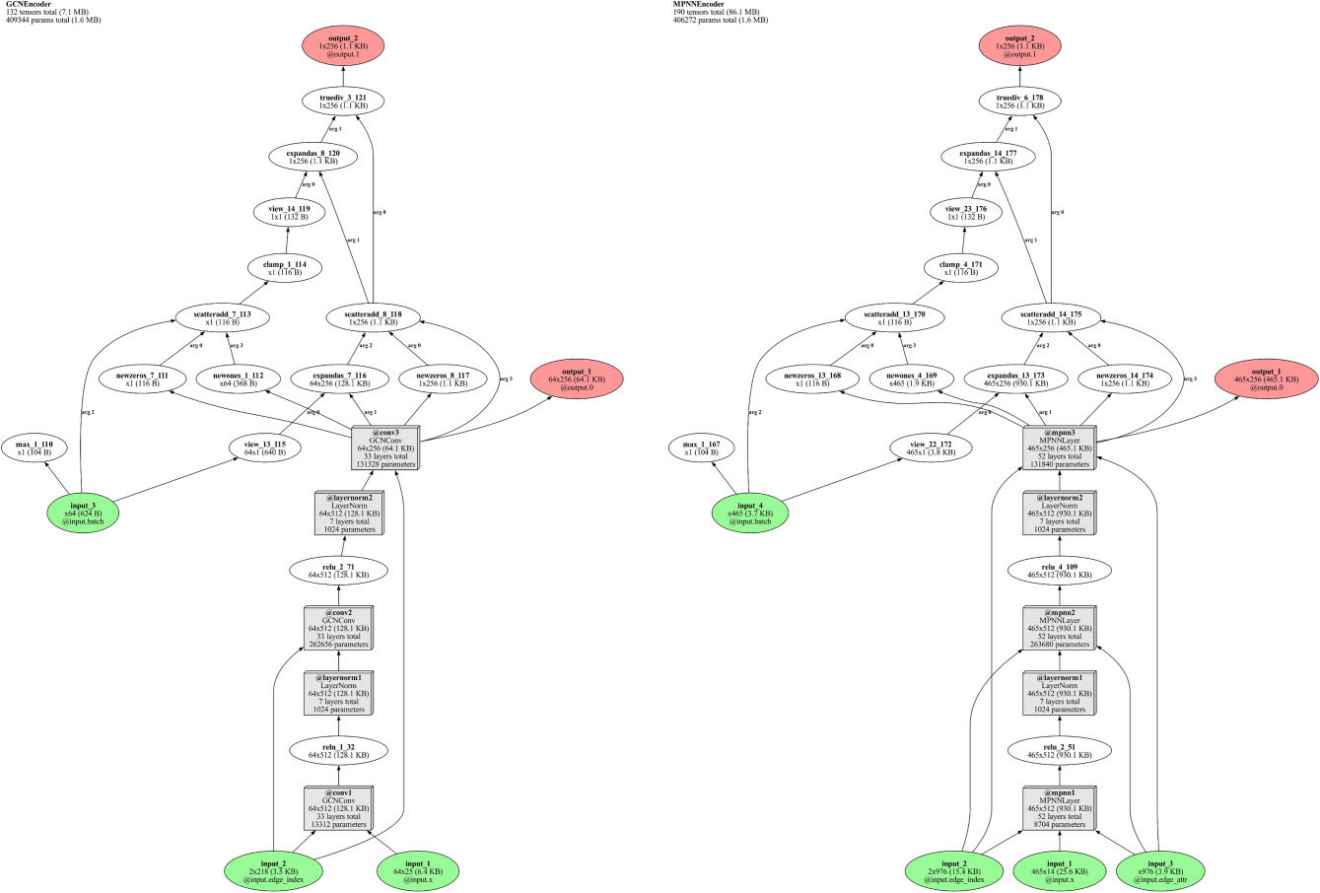
Schematic representation of the GCEncoder and the MPNNEncoder, created using torchlens [35].
- To accommodate atomic-scoped graphs and incorporate edge features, the simple GCN layers in the GCEncoder were replaced with message passing neural network (MPNN) layers, resulting in a new encoder, the MPNNEncoder. The MPNN layers consisted of a GCN for node features and a linear layer for edge features, facilitating the transformation and integration of both node and edge information, which enhanced the model’s ability to capture more intricate graph structures. Like the GCEncoder, for each MPNN layer a ReLU activation was applied followed by layer normalization, except for the final one. The node embeddings from the final MPNN layer were pooled to achieve a graph-level embedding. To create graph embeddings of dimension 128, two MPNN layers were used; for embeddings of dimension 256, three MPNN layers were used.

- Two GAEs were designed, one for each graph dataset. Both architectures were implemented using PyTorch Geometric [33], each composed of distinct custom encoders and the same inner product decoder [29]. The schematic architecture of both autoencoders can be seen in Fig. 3. The graph convolutional encoder (GCEncoder), designed to handle residue-scoped graphs, comprised graph convolutional network (GCN) layers, each followed by a layer normalization applied per graph. A rectified linear unit (ReLU) activation function was applied after each GCN layer, excluding the final one. The node embeddings from the final GCN layer were pooled using a global mean pooling operation to obtain a graph-level embedding. To create graph embeddings of dimension 128, two GCN layers were used; for embeddings of dimension 256, three GCN layers were used.

Irrespective of the encoder type, an inner product decoder was utilized to decode the node embeddings, or latent variables, into edge probabilities and a probabilistic dense adjacency matrix. Both GAEs were trained to minimize the binary cross-entropy loss for positive edges and negative sampled edges. Thus, the reconstruction loss was calculated as the sum of the losses for positive and negative edges. The GAEs were trained over a maximum of 20 epochs using a batch size of 32 and a learning rate of 0.005 with the Adam optimizer. Early stopping was implemented and evaluated epoch wise. The decision to apply early stopping was based on validation accuracy with a patience latency of three epochs without an increase in validation accuracy. The training, validation, and test data were loaded using PyTorch’s DataLoader [33], which provided the data in mini batches during training. After training, the models were tested using the test data, and the area under curve (AUC) and average precision scores were reported. Upon completion of training, the entire graph datasets, representing all predicted protein structures (wild-type and variant structures), were processed through the trained GAEs. This step enabled us to extract numerical embeddings for both the variant and wild-type structures across the entire datasets. These embeddings provide a condensed yet comprehensive representation capturing both local and global information of the protein structures, serving as a crucial input for subsequent analysis and training of an XGBoost classifier [30]. The pooling operation utilized in this approach certainly comes with its limitations as discussed by Gu *et al.*[36], highlighting that applying a global mean pooling operation, which is an approach to transform the individual node embeddings into graph-wide embeddings, probably leads to large loss of information. To reduce the information loss for the residue-scoped experiments, next to the aggregated embeddings, we also extracted the node embeddings of the wild-type and variant structure at the specific protein position where the amino acid exchange is happening to preserve more of the local context caused by the exchange. These node embeddings were used as a separate feature in the residue-scoped experiments.

### Setting up a five-fold cross validation and an external test dataset

We created an external dataset, which was set aside during training and validation (as well as hyperparameter tuning) and was purely used for the final evaluation of the pathogenicity predictors. At first, we planned to use one of the ClinVar datasets from the publication of Pejaver *et al.*[37] in which they utilized these datasets to calibrate multiple *in silico* pathogenicity predictors. However, ProteinGym has a large overlap with those datasets. Therefore, we decided to compute the overlap between ProteinGym and the ClinVar 2020 dataset from the aforementioned publication. Subsequently, we utilized UniProt to match the individual proteins from this overlap dataset to their protein families to account for sequence similarity between those overlapping sets and filtered out every protein from ProteinGym that belonged to a similar protein family as those proteins present in the ClinVar 2020 dataset and added them to the hold-out dataset. This step was crucial to account for possible data leakage resulting from highly similar proteins in both datasets as described by Bernett *et al.*[38]. This final test dataset contained 12 590 variants from 440 unique proteins from 95 protein families. Five thousand eight hundred eighty-five variants were assigned the label benign, and 6705 variants were assigned the label pathogenic. The remaining non-overlapping ProteinGym dataset consisted out of 47 399 variants from 1966 unique proteins. Subsequently, this remaining ProteinGym dataset was split into five folds, which were eventually used to set up a five-fold cross validation. To prevent intergenic data leakage, we ensured that variants of genes present in one fold did not occur in another fold, which could have resulted in the problematic situation that variants from the same gene would end up in training, validation, evaluation sets, and the hold-out test set. This step was critical to ensure that our model’s performance evaluation was accurate and not influenced by any overlapping data between the training, validation, and testing phases. While the five folds were equally sized in terms of genes per fold, genes contained in the ProteinGym clinical substitution dataset don’t contain the same number of variants, and as previously mentioned, benign and pathogenic variants are not equally distributed in the ProteinGym either. However, this led to only a slight class imbalance in the folds, which therefore did not require further resampling.

### Implementation and training of a XGBoost classifier

To predict the pathogenicity of missense single nucleotide variants, we adopted an XGBoost classifier [30] using the XGBoost library, a gradient boosting framework renowned for its predictive accuracy and computational efficiency. For the training, validation, and evaluation, we used a five-fold cross validation. Subsequently, an XGBoost model was trained and evaluated five times using the prepared folds. For each training setup an individual data split was performed. The training set contained three folds, and the validation as well as the evaluation set contained one fold. The hyperparameters of the classifier were optimized using Optuna [39], a hyperparameter optimization framework. The Optuna [39] hyperparameter optimization was conducted over 100 trials to determine the optimal set of hyperparameters for the XGBoost [30] model. The performance metric for the optimization was the accuracy of the model, deployed on the evaluation set of the current fold. This optimization was performed individually for each fold split. Subsequently, the five sets of tuned hyperparameters were averaged and used to train a meta-optimized model on the fivefold splits, which was used to evaluate the final performance of the classification model on the hold out test set, which was not seen prior to this point by any classifier. This procedure was performed three times using different feature combinations, each with consistent fold splits. In the first experimental setup, the dataset consisted of various combinations of the pooled graph embeddings, the node embeddings, and the cosine distance between the wild type and variant embeddings, both on the level of the pooled graph embeddings and the individual node embeddings. The target variable was the pathogenicity of the variants, encoded into numerical form. As reference, we calculated the performance metrics using only the CADD score (v 1.7) for classification, utilizing the suggested threshold for binary filtering (CADD ≥ 20). We repeated this experiment with the same setup, with the exception of the inclusion of the CADD score (v 1.7) as a feature, to examine the added effectiveness of three-dimensional information on a widely applied existing pathogenicity prediction score. Evaluation criteria were the area under the curve of the receiver operating curve (AUROC), the precision-recall curve, the confusion matrix, sensitivity, specificity, the positive predictive value, the negative predictive value, and the Matthews correlation coefficient (MCC) [40]. Models, their hyperparameters, and their performance metrics were logged and stored using MLflow [41], a platform for managing the machine learning lifecycle. This approach allowed us to efficiently manage, track, and evaluate the performance of our machine learning models. The averaged hyperparameters for each experiment can be found in the supplementary information, attached to this article as an Excel table.

### Computation of Shapley values

To understand the feature importance and overall model impact per feature in our XGBoost classifiers, we computed the Shapley values (SHAP values). SHAP (SHapley Additive exPlanations) [42] values are a useful tool for understanding which features influence a model’s predictions the most. Grounded in cooperative game theory, SHAP values provide a detailed breakdown, showing how changes in each feature affect the model’s output, allowing to examine which features are driving predictions. The SHAP values were calculated using the aggregated classifiers for the hold-out test set. These values were then stacked across the hold-out dataset to assess the overall feature importance. By default, the SHAP values are computed for each input feature, which in our case corresponded to each element in our structural embeddings. Given the additive nature of the SHAP values, we wanted to consider the structural embeddings as a whole. To achieve this, we summed the SHAP values over each structural embedding space. This allowed us to obtain a single SHAP value per structure embedding, providing a comprehensive understanding of the importance of the entire structural embedding in the model. These steps allowed us to create feature importance plots, which visually represented the significance and total impact of each feature in the model.

## Results

### Pathogenicity prediction

**Figure 4.**
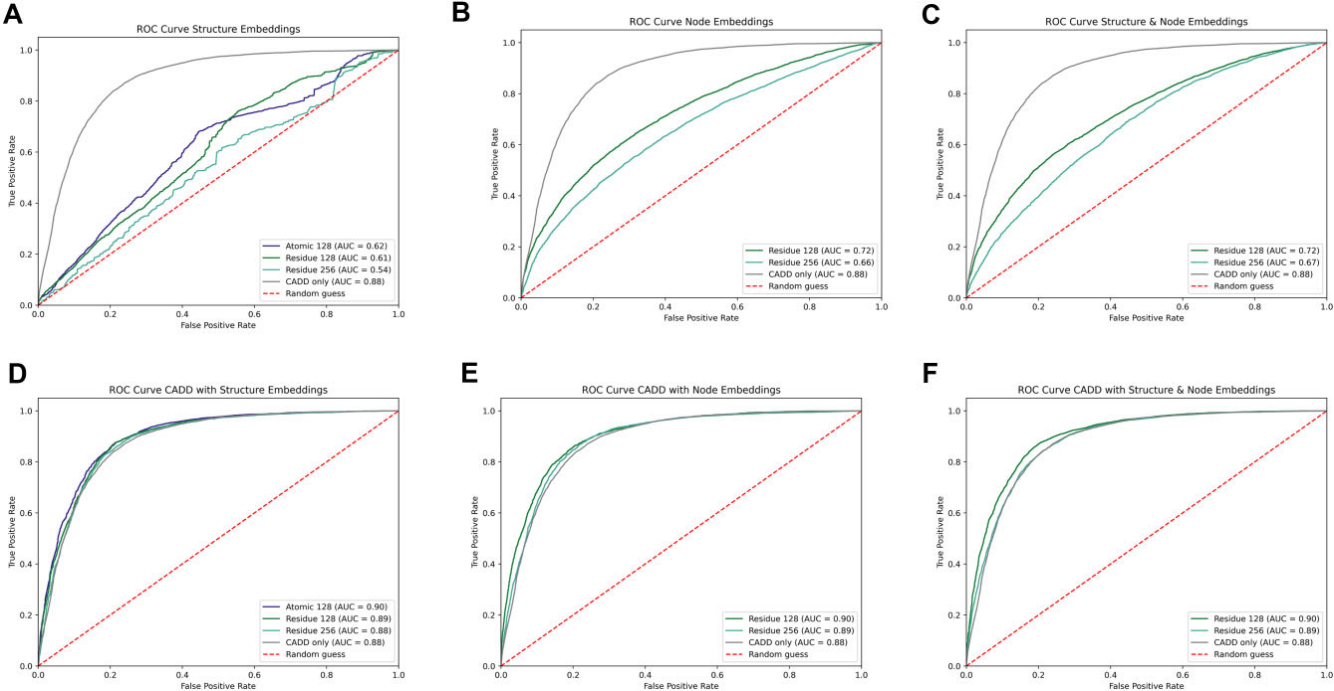
Comparison of classifier performance using various input features for pathogenicity prediction of missense variants. (**A**) ROC curves for classifiers trained exclusively on pooled graph embeddings (purple, green, cyan) versus CADD score alone (grey). (**B**) ROC curves for classifiers using only node embeddings (green, cyan) compared to the CADD score (grey). (**C**) ROC curves for classifiers trained with a combination of pooled graph embeddings and node embeddings (green, cyan) versus the CADD score baseline (grey). (**D**) ROC curves for classifiers incorporating both pooled graph embeddings and the CADD score (purple, green, cyan), compared to the CADD score alone (grey). (**E**) ROC curves for classifiers trained with node embeddings and the CADD score (green, cyan) versus the CADD score baseline (grey). (**F**) ROC curves for classifiers using the CADD score in combination with both pooled and node graph embeddings (green, cyan), compared to the baseline CADD score (grey).

To access the value of *in silico* predicted three-dimensional protein structures for pathogenicity prediction of missense variants, we trained multiple XGBoost [30] classifiers. Each model incorporated structural graph embeddings at various abstraction levels, with some also receiving the CADD score as an additional input feature. Model performance was evaluated using the AUROC metric, confusion matrices, precision-recall curves, sensitivity, specificity, accuracy, and the MCC. Figure 4 displays the ROC curves for all classifiers, while Table 1 summarizes AUROC, MCC, and accuracy (additional metrics are provided in the supplementary material). Additionally, Supplementary Fig. S1 depicts the precision recall curves of the binary classifiers. Across all evaluated metrics, classifiers relying solely on graph embeddings underperformed compared to those that included the CADD score or the CADD score alone (Fig. 4A–C). Notably, models using only pooled graph embeddings showed substantially lower AUROC scores than those using only node embeddings corresponding to the mutated amino acid, a trend consistent across all abstraction levels. (Fig. 4 A and B). Models combining node and pooled embeddings produced mixed results, with only slight variations in AUROC, MCC, and accuracy.

**Table 1.** Performance summary of the binary classifiers

Experiment	ROC AUC	MCC	Accuracy
CADD only	0.882 542	0.536 982	0.745 353
Residue 128 CADD with node embeddings	0.900 165	0.658 207	0.829 944
Residue 256 CADD with node embeddings	0.888 499	0.643 314	0.822 399
Atomic 128 CADD with structure embeddings	0.898 144	0.657 425	0.829 627
Residue 128 CADD with structure embeddings	0.891 255	0.636 924	0.81 811
Residue 256 CADD with structure embeddings	0.884 693	0.642 209	0.821 843
Residue 128 CADD with structure & node embeddings	0.902 431	0.642 323	0.819 619
Residue 256 CADD with structure & node embeddings	0.885 741	0.63 286	0.817 315
Residue 128 node embeddings	0.723 043	0.32 771	0.655 679
Residue 256 node embeddings	0.662 061	0.234 528	0.614 138
Atomic 128 structure embeddings	0.615 437	0.134 888	0.542 335
Residue 128 structure embeddings	0.606 924	0.096 241	0.523 987
Residue 256 structure embeddings	0.544 299	0.05 941	0.515 806
Residue 128 structure & node embeddings	0.719 549	0.311 119	0.628 197
Residue 256 structure & node embeddings	0.671 452	0.213 437	0.583 082

The best structure-only classifier integrated node embeddings at the residue level with an embedding size of 128. Although graph embeddings alone lagged behind CADD on the holdout test set, combining structural embeddings with the CADD score consistently outperformed the CADD score by itself (Fig. 4D–F and Table 1). The highest AUROC was achieved by a model incorporating CADD alongside both node and pooled residue-level embeddings (embedding size 128), while MCC and accuracy peaked for the model combining CADD with only node embeddings at the same level and size. Both combined models outperformed CADD alone across all three metrics, with the most prominent gains observed in MCC and accuracy.

When compared to their residue counterpart, the model integrating pooled graph embeddings on the atomic level of abstraction with an embedding size of 128 showed comparable performance in terms of AUC, MCC, and the accuracy score. However, it was outperformed when compared to the residue-scoped model integrating both the pooled graph embeddings and the node-level embeddings. Finally, we examined the impact of embedding dimension at the residue level, finding that models with an embedding size of 128 consistently outperformed those with the larger size of 256 across all evaluated metrics.

### Feature importance

**Figure 5.**
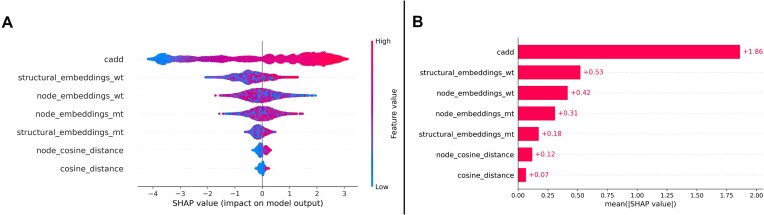
SHAP values displayed as a bee swarm plot (**A**), highlighting the individual SHAP values. Additionally, the aggregated SHAP values are displayed as a bar plot (**B**). The CADD score is the undisputed most important feature. The graph embeddings are of noticeable importance. The wild-type structures are ranked as more important to the classification task compared to the variant structures. The node embeddings are of remarkable importance, despite the inclusion of the pooled structure embeddings.

To further explore the relevance of graph embeddings for the classification task, we utilized the aggregated XGBoost classifiers at the residue level of abstraction (embedding size = 128) to predict the SHAP values for the hold-out test data. We aggregated these SHAP values for an overall evaluation, which can be seen in Fig. 5. As visualized in Fig. 5, the CADD score is the most influential feature for pathogenicity prediction, which is then followed by the pooled structural embeddings of the wild-type structures, the node embeddings of the wild-type structures, the node embeddings of the variant structures, the pooled embeddings of the variant structures, and finally both cosine distances.

### Evaluating the impact of the integration of protein graph embeddings on different protein families

**Figure 6.**
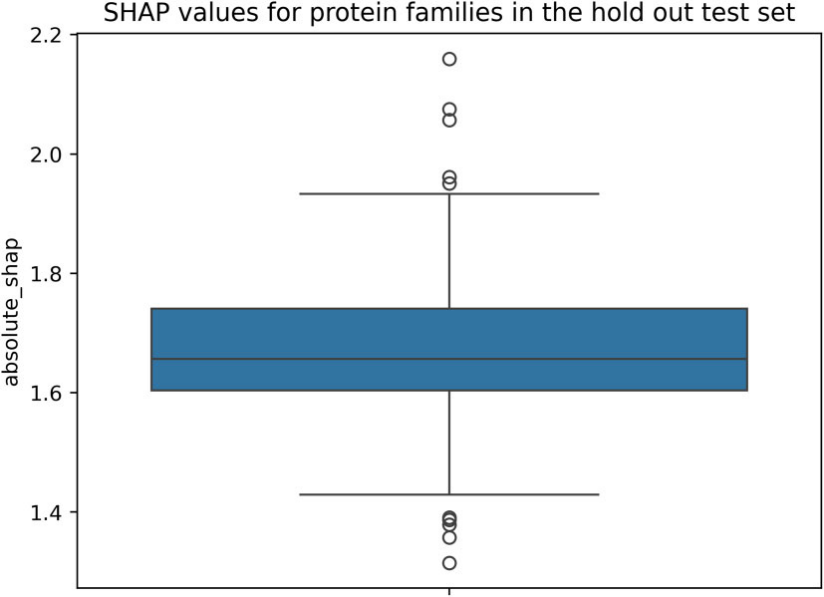
Boxplot depicting the mean absolute SHAP values calculated for the 95 protein families present in the test dataset.

To further explore the generated SHAP values, we aggregated the absolute SHAP values from the pooled graph embeddings and the node embeddings per variant and averaged these sums across the previously mentioned 95 protein families obtained from UniProt. Proteins that were not matchable using the UniProt ID mapping procedure were dropped during this process. Across these 95 protein families we observed a mean absolute SHAP value of 1.66 with 0.18 as a standard deviation and a median absolute SHAP value of 1.63. A boxplot depicting these absolute SHAP values for the protein families can be seen in Fig. 6.

### Comparing graph embeddings from different protein folding models

**Figure 7.**
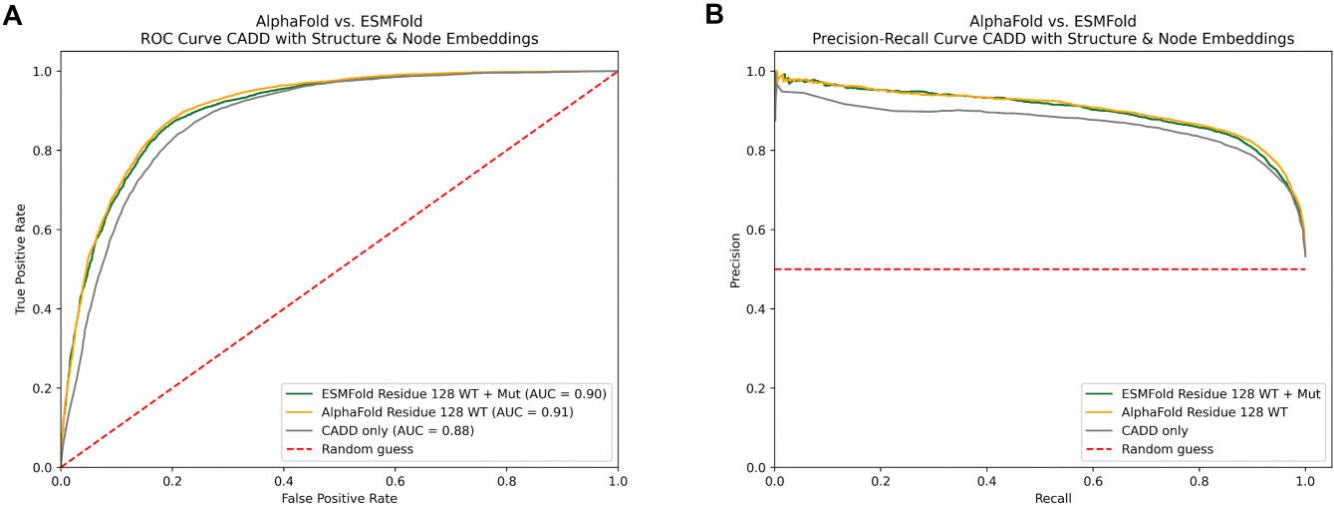
(**A**) ROC curves comparing classifiers trained on the CADD score combined with graph embeddings (residue-level abstraction, embedding size 128) obtained from a GAE trained on ESMFold structures. Classifiers using embeddings derived from AlphaFold structures are shown in yellow, while those using ESMFold embeddings are depicted in green. The grey line indicates the CADD score baseline. (**B**) Precision-recall curves for the same classifiers. Yellow represents the use of AlphaFold embeddings, green denotes ESMFold embeddings, and grey shows the CADD baseline.

While most of the analysis in this manuscript was focused on *in silico* structures predicted by ESMFold, we hypothesized that the presented workflow is agnostic towards the source of protein structures. To put this hypothesis to the test, we obtained wild-type structures predicted by AlphaFold2 for the proteins present in ProteinGym. Due to the limited availability of precomputed AlphaFold2 structures for variants, we limited ourselves to only make use of the wild-type structures. We embedded these AlphaFold2 structures using the autoencoder model, trained on ESMFold data. Subsequently, we repeated the experiments (with residue level of abstraction and embedding size of 128), differing that only either ESMFold or AlphaFold2 wild-type structures were used to augment the CADD score. The resulting ROC curve, the precision-recall curve, and the comparison to the previously best-performing model can be seen in Fig. 7. The classifier integrating structural embeddings from AlphaFold outperformed all classifiers integrating structural information from ESMFold and therefore was the overall best-performing model, despite only including graph embeddings from wild-type structures. A performance comparison of these classifiers can be found in Table 2.

**Table 2.** Performance summary for the comparison between a classifier trained on AlphaFold and ESMFold data

Experiment	ROC AUC	MCC	Accuracy
CADD only	0.882 542	0.536 982	0.745 353
ESMFold residue 128 WT + Mut with structure & node embeddings	0.902 431	0.642 323	0.819 619
AlphaFold residue 128 WT CADD with structure & node embeddings	0.907 859	0.66 095	0.830 016

## Discussion

### Differentiating to existing models

The demonstrated workflow presents a way to include the three-dimensional structure of proteins in pathogenicity classification tasks. In principle this agnostic workflow is capable of processing experimentally determined and *in silico* predicted structures. Models like AlphaFold2 [20] and ESMFold [24] have made *in silico* predicted protein structures abundantly available; however, these structures have only been partially used in variant effect predictors previously. Previous structural-aware models, like those presented by Schmidt *et al.* and Zhao *et al.*, take structural information from *in silico* predicted protein structures from AlphaFold2 into account; however, they rely on an engineered feature extraction process in which biochemical and network features are extracted and then used to train a classifier model [21, 22]. The presented approach differentiates itself from those previous workflows by replacing the manual feature engineering process with graph embeddings generated by GAEs, which are compatible with *in silico* predicted structures from arbitrary computational modelling approaches and real-world structures.

### Exploring protein graph embeddings at different levels of abstraction

We examined whether the level of abstraction used to transform *in silico* generated protein structures to protein graphs has different effects on a classifier used for pathogenicity prediction. The best-performing model in our initial experiments was a model trained on structural embeddings on the residue level of abstraction with an embedding size of 128. This could have different explanations. Although it has been demonstrated that variant structures from protein-folding models contain useful information that can be leveraged in downstream tasks [21, 22], protein-folding models still fall a little bit short when evaluated on the atomic scope [43–46]. We therefore hypothesize that the most likely explanation in our case would be that embeddings generated from structures generated by the ESMFold model on the atomic scope do not accurately capture actual atomic constellations. This effect is probably further amplified in variant structures on the atomic scope, because, as introduced, they already provide difficulties for current models. Furthermore, we had to limit the encoding for the atomic graphs to covalent bonds as edges due to computational limitations, resulting in an information loss. Additionally, the generated graph embeddings at the atomic level of abstraction exhibited sparsity compared to those at the residue level of abstraction. Future work is needed in which this is explored in more detail, possibly even in a comparison between *in silico* and real-world structures and using different embedding strategies. It might be worth mentioning that geocoordinates provide spatial orientation information. However, a disadvantage is that adding these coordinates renders the model non-invariant to Euclidean transformations. In our experiments, we ensured that both the wild-type and variant structures were not subject to translation or rotation. Without incorporating the 3D coordinates, the autoencoders would remain invariant to Euclidean transformations, which offers a significant advantage. The impact of this choice on model performance is to be the subject of further experiments to fully understand its implications.

### Comparing protein graph embeddings from different sources

Due to computational limitations, we opted to use the 3 billion parameter ESMFold model, available on Hugging Face [31]. Because of the faster inference and smaller computational requirements when compared to AlphaFold2, we were able to predict the wild-type and variant structures for the whole ProteinGym clinical substitution dataset. This, however, is a possible entry point for a performance ceiling effect, since for ESMFold it has been demonstrated that an increase in parameter size results in more accurate predictions. Additionally, since its release, ESMFold has been described as slightly less accurate compared to AlphaFold2 [24]. This reduced accuracy might be inherited by the presented classifiers and therefore should be kept in mind as potential limitation. As previously introduced, we hypothesize this workflow to be somewhat source agnostic. We provide evidence for this protein structure agnosticism with our AlphaFold2 experiment by demonstrating that a classifier trained with AlphaFold2 graph embeddings (generated using an autoencoder trained purely on ESMFold data) achieves similar performances when compared to a classifier trained on ESMFold graph embeddings (generated using an autoencoder trained purely on ESMFold data). Because of limited computational resources, we did not have the capacity to additionally fold all variant structures utilizing AlphaFold2 or even AlphaFold3 [47], which would be an interesting comparison to be made in a future project. We justify this shortcut by information obtained from Fig. 5. While both types of structural embeddings (wild type and variant) influence the model’s performance, the wild-type structures seem to have a larger impact. This larger impact was observable across all our experiments. Therefore, we estimated that the wild-type structures alone can be used to obtain evidence for generalizability across different structures from different sources. This is supported by the model trained on AlphaFold structures, as it was the overall best-performing model, despite only being presented with wild-type structures during training. Yet, it clearly demonstrates the flexibility of this method by allowing the integration of protein structures from different sources. In future work, this idea needs to be examined by performing experiments containing wild-type and variant structures from different sources.

### Protein structures as a utility in existing pathogenicity prediction classifiers

Additionally, we provide evidence that protein graph embeddings can enhance existing pathogenicity predictors, using the CADD score (v 1.7) as an example. Our results show that integrating structural embeddings improves the performance of the CADD score on the hold-out test set, as measured by various performance metrics. In its version 1.7, the CADD score already incorporates predictions from the ESM protein language model. However, we observed a performance improvement when combining it with protein graph embeddings. This suggests that the folding component of the ESMFold model generates novel information not prominently preserved in the language model’s component. This observation supports the value of integrating structurally derived information from *in silico* structures. The CADD score is a widely applied pathogenicity prediction metric that is precomputed for the whole human genome. Since the AlphaFold database provides access to a large variety of precomputed *in silico* structures, the presented approach can be applied to a large variety of variants falling into the coding region of the human genome. While recent predictors such as AlphaMissense [6], EVE [48], REVEL [7], and others have demonstrated strong performance, often by integrating diverse sources of information including population allele frequencies, our present goal was not to propose a new state-of-the-art pathogenicity classifier, but rather to demonstrate the measurable gains from explicitly adding structural protein information to existing frameworks. We selected the CADD score for this project, as it is widely used, genome-scale, and—crucially for our study design—already includes sequence-based ESM protein language model embeddings. Our results indicate that even with the inclusion of advanced protein sequence features, integrating explicit 3D structural information via graph embeddings yields further improvement. Thus, incorporating protein structure embeddings into existing pathogenicity scores such as the above mentioned or combining such structural features with multiple predictors in an ensemble could be a valuable direction for future research to further improve variant effect prediction.

### Analysis of feature importance using SHAP values

As visible in Fig. 5, the CADD score is the undisputed most important feature in the presented classifier. Yet, the protein graph embeddings are of clear feature importance. The SHAP values demonstrate that the integration of the node-level embeddings is a reasonable approach to compensate the loss of information caused by the global mean pooling operation, which is performed to obtain the aggregated graph-level embeddings. Future work in which additional architectures and pooling strategies are explored is necessary. Additionally, as previously introduced, the SHAP values demonstrate that the presented classifiers are less influenced by the protein graph embeddings from the variant structures. This again can have multiple causes. It might be explainable by the possible overlap of information between wild-type and variant structure embeddings. Furthermore, this might indicate the difficulty the ESMFold model faces in generating variant structures, suggesting that future experiments should reproduce these with varied combinations of variant and wild-type structures obtained from different protein language models. Finally, we utilized the SHAP values from the variants present in the test set to evaluate the hypothesis of whether individually protein families benefit more than others from the integration of protein graph embeddings. However, with the exception of the outliers visible in Fig. 6, we observed a relatively narrow distribution of absolute SHAP values across the different families, which might suggest a quite uniform benefit across the protein families present in the test set. To further explore this hypothesis larger datasets with the integration of a diverse collection of protein families are necessary.

## Summary

We developed a machine learning workflow that can generate graph embeddings from protein structures, which can be used in downstream tasks. We explored the utility of these embeddings in the application case of pathogenicity prediction of missense variants. We explored different levels of abstraction in these graph embeddings, compared *in silico* structures from various sources, and evaluated the impact of combining the existing CADD pathogenicity prediction score with these graph embeddings. Our experiments highlight the additional value of these graph embeddings, demonstrating that they provide information not encapsulated in current pathogenicity prediction models. The presented work primarily serves as a machine learning workflow with the aim to further establish the integration of *in silico* predicted structures for downstream tasks like variant prioritization. However, in principle, the generated protein embeddings could also be applied to other research questions, such as protein function prediction, thereby opening a wide range of applications.

## Supplementary Material

lqaf097_Supplemental_Files

## Data Availability

The code and sample data to reproduce the workflow are available in the GitHub repository: https://github.com/IHGGM-Aachen/genoseer and Zenodo: https://zenodo.org/records/15723477. The repository is also attached to this article as supplementary data.
