# Supplementary material for: Utilizing protein structure graph embeddings to predict the pathogenicity of missense variants
Source: NAR Genom Bioinform. 2025 Jul 24;7(3):lqaf097. doi: 10.1093/nargab/lqaf097 (PMC12288871; doi:10.1093/nargab/lqaf097)
Supplement: lqaf097_Supplemental_Files [file lqaf097_supplemental_files.zip › Supplementary_Information_Revision.docx]

**Supplementary Information for the article:” Utilizing protein structure graph embeddings to predict the pathogenicity of missense variants”**

Martin Danner^1,2^, Matthias Begemann^1^, Miriam Elbracht^1^, Ingo Kurth^1^, Jeremias Krause^1^

^1^Institute for human genetics and genomic medicine, Medical Faculty, Uniklinik RWTH Aachen, Pauwelsstrasse 30, Aachen, 52074, North-Rhine-Westphalia, Germany.

^2^scieneers GmbH, Kantstraße 1a, Karlsruhe, 76137, Baden-Wuerttemberg, Germany.

Correspondence should be addressed to:

Jeremias Krause

Institute for Human Genetics and Genomic Medicine

Medical Faculty, RWTH Aachen University

Pauwelsstrasse 30

D-52074 Aachen

Tel.: +49 241 87012

E-Mail: [jerkrause@ukaachen.de](mailto:jerkrause@ukaachen.de)


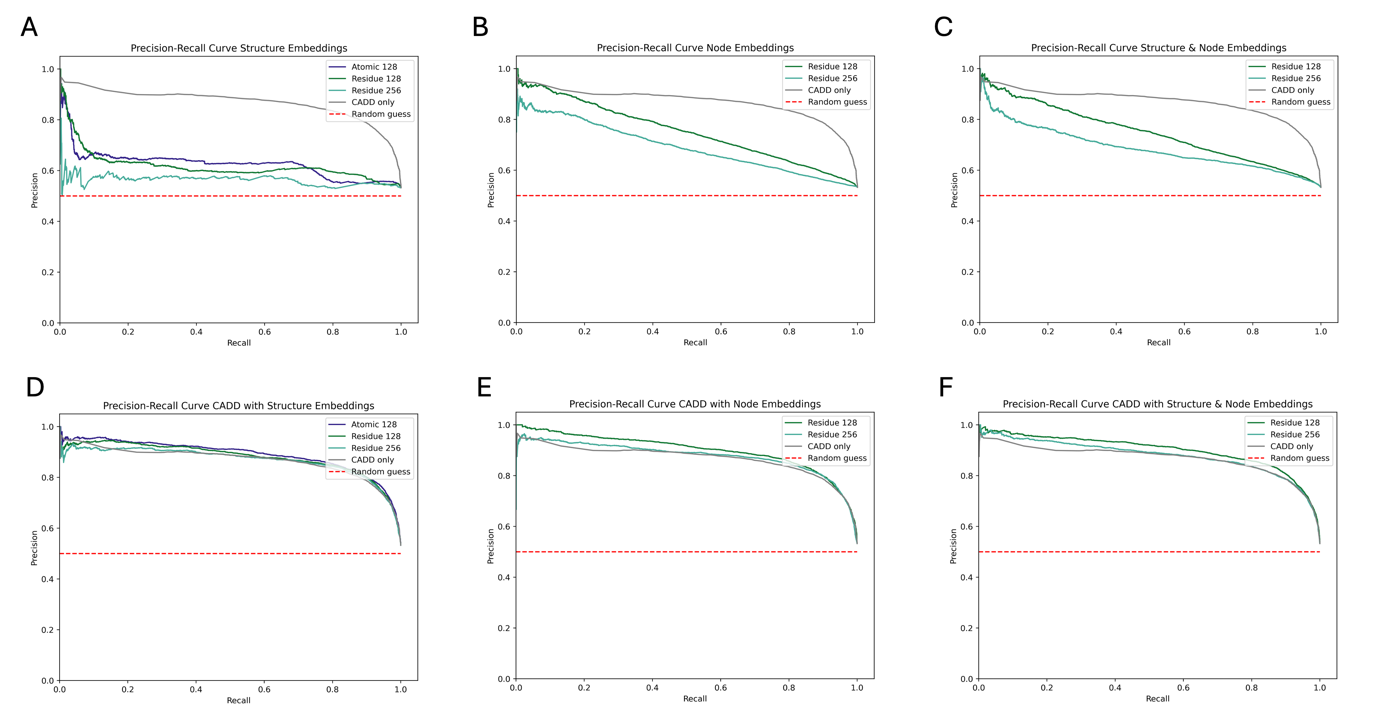


Supplementary figure 1: Side-by-Side comparison of the performance of the different classifier models. (A) Precision-Recall-Curves of Classifiers trained solely with the pooled graph embeddings (purple, green and cyan) compared to the CADD score as baseline (grey). (B) Precision-Recall-Curves of Classifiers trained with solely the node embeddings (green and cyan) compared to the CADD score as baseline (grey) (C) Precision-Recall-Curves of Classifiers of Classifiers trained with the combination of the pooled graph embeddings and the node embeddings (green and cyan) compared to the CADD score as baseline (grey) (D) Precision-Recall-Curves of Classifiers trained with the pooled graph embeddings and the CADD score (purple, green and cyan) compared to the CADD score as baseline (grey). (E) Precision-Recall-Curves of Classifiers trained with the node embeddings and the CADD score (green and cyan) compared to the CADD score as baseline (grey). (F) Precision-Recall-Curves of Classifiers trained with the CADD score and the combination of the pooled graph embeddings, and the node embeddings (green and cyan) compared to the CADD score as baseline (grey)
